# Epidemiological Study of Pathogenic *Leptospira* in Raccoons (*Procyon lotor*) in a Suburb of Tokyo, Japan

**DOI:** 10.3390/ani13010021

**Published:** 2022-12-21

**Authors:** Kazuki Kiuno, Takuya Kato, Hiroko Otsubo, Ryoko Kibe, Yasushi Kataoka, Shin-ichi Hayama

**Affiliations:** 1 Laboratory of Veterinary Wildlife Medicine, Nippon Veterinary and Life Science University, 1-7-1 Kyonan-Cho, Musashino-Shi, Tokyo 180-8602, Japan; 2 Laboratory of Veterinary Microbiology, Nippon Veterinary and Life Science University, 1-7-1 Kyonan-Cho, Musashino-Shi, Tokyo 180-8602, Japan

**Keywords:** *Leptospira*, Raccoon, Urban, Dispersal, Environmental factor

## Abstract

**Simple Summary:**

Leptospirosis is a zoonotic infection, meaning that the pathological agent is transmitted between animals and humans. Raccoons are known to be one of the vectors, and there is an increasing concern regarding the spread of pathogens in Japan with expanding distribution and population of raccoons. We detected leptospiral DNA and antibodies in captured raccoons, and the prevalence and seroprevalence rates differed depending on the dispersal period of raccoons, showing that young males may spread *Leptospira* with dispersal. Furthermore, raccoons were found to contain *Leptospira* during warm and rainy seasons, which are suitable for the survival of *Leptospira* and wild rodents. Raccoons in urban environments may be more exposed to *Leptospira*.

**Abstract:**

Leptospirosis is a zoonosis that affects humans and animals worldwide. Raccoons (*Procyon lotor*), adopted in urban environments, may act as potential reservoirs of *Leptospira*. We investigated the prevalence of pathogenic *Leptospira* in the kidney and urine samples of raccoons living in Tokyo, as well as anti-leptospiral antibodies in their serum, and aimed to examine the factors that expose raccoons to *Leptospira*. Polymerase chain reaction (PCR) and enzyme-linked immunosorbent assay (ELISA) were used to detect leptospiral DNA and anti-leptospiral antibodies, respectively. Thirty-six of 156 raccoons (23.1%) were positive by PCR, and 16 of 165 raccoons (9.7%) were positive by ELISA. The prevalence and seroprevalence rates differed depending on the raccoon dispersal period. We used univariable logistic regression to estimate the environmental factors associated with pathogenic *Leptospira* and anti-leptospiral antibodies in raccoons. Significant differences were observed in the PCR results for the seasons (spring–summer) (*p* = 0.01), average monthly temperature (*p* < 0.01), and average monthly rainfall (*p* < 0.01). No significant difference was seen in the ELISA results, but raccoons in larger urban areas tended to have higher seroprevalence rates (*p* = 0.06). We identified a pattern of leptospiral spread in raccoon dispersal and environmental factors that expose raccoons to *Leptospira*.

## 1. Introduction

Leptospirosis is a zoonosis caused by pathogenic *Leptospira* (*Leptospira interrogans* [discovered by Stimson in 1907] sensu lato) [1]. Most mammals can be infected with *Leptospira* and have been implicated as potential vectors [2]. Many infected mammals, including wild rodents playing an important role as reservoirs, excrete *Leptospira* in their urine and are infected transdermally or transmucosally through direct contact with the urine or indirect contact with water or soil contaminated with urine [3,4]. Outbreaks of leptospirosis in humans occur worldwide, including those reported in tropical areas, North America, and Europe, and have also been reported throughout Japan, especially in Okinawa Prefecture [5,6]. In recent years, there has been a growing risk of public health-related problems in urban environments owing to climatic changes such as global warming and heavy rainfall [7,8].

Raccoons (*Procyon lotor* [Linnaeus 1758]), which are native to North America, can also get infected with *Leptospira* and become its vector [9,10]. Raccoons were first established in Japan in Aichi Prefecture in 1962, and wild raccoon breeding has now been confirmed in 46 prefectures [11,12]. In recent years, the distribution and population expansion of raccoons have become a serious problem in many parts of Japan [13]. In the Kanto region, which is located in the center of Japan and includes the mega-city Tokyo, the number of captured raccoons continues to increase year by year, and there is a growing concern about their distribution and population expansion [14]. In Tokyo, the number of captured raccoons, designated as a specified invasive alien species, has been on the rise owing to severe agricultural damage caused by raccoons, and there is concern that their distribution and population will expand [15]. The expansion of raccoon distribution and population increases agricultural damage and raises concerns about the transmission of zoonosis in humans and animals [12].

Raccoons show a subclinical infection with *Leptospira*, suggesting that they act as subclinical reservoirs [16]. Leptospiral DNA and antibody detection cases have been reported in raccoons in Japan [17,18,19,20,21]. Leptospiral DNA has been detected in approximately 10% of raccoons in Osaka and Hyogo and 23.2% of raccoons in Hokkaido [18,20,21]. *Leptospira* have been isolated from the kidneys of raccoons living in Kanagawa Prefecture [17]. This suggests that raccoons may spread *Leptospira* as their distribution and population expand and may have a new role in the related infection cycle in Japan. Raccoon dispersal behavior has been shown to vary with sex and age [22,23]. As dispersal behavior at different distances and periods may affect the opportunities related to the exposure to pathogens present in the environment, leptospiral DNA and antibody detection status may also be influenced by sex and age.

*Leptospira* in raccoons are related to raccoon population density and the urban environment [21,24,25]. In addition, the water environment is considered a potential site for exposure of raccoons to *Leptospira* because raccoons spend much of their time foraging in water environments [26]. Their environment may play an essential role for raccoons to contact *Leptospira*, and no studies have quantified and estimated the environmental factors underlying the relationship between raccoons and *Leptospira*. The estimation of environmental factors can lead to the analysis of areas at a high risk of infection, which may be important for controlling the spread of disease.

In this study, we detected pathogenic leptospiral DNA and anti-leptospiral antibody in raccoons living in a suburban area of Tokyo, Japan. We examined the factors that lead to the exposure of raccoons to *Leptospira*.

## 2. Materials and Methods 

### 2.1. Sample Collection

**Figure 1:**
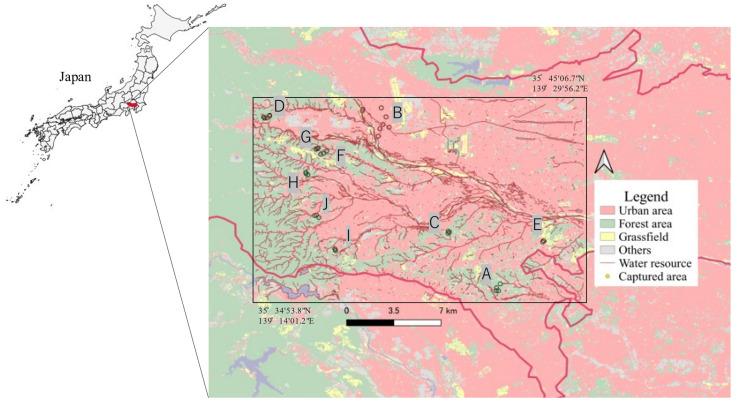
Map of the Tama area, Tokyo, Japan. The study area is marked by the rectangle.

Raccoons were captured between June 2020 and April 2022 in 10 areas, including residential areas (point B) and green areas around urban areas (point A, C–I) within the Tama region of Tokyo. The study area is illustrated in Figure 1. Raccoons were captured as part of the “Tokyo Metropolitan Government Raccoon and Masked Palm Civet (*Paguma larvata*) Control Implementation Plan [15]” and “Pest control by municipalities”. Raccoons were captured using box traps by licensed hunters, veterinarians, or personnel authorized by the local government. The captured raccoons were euthanized following the Guidelines for the Management of Invasive Alien Species of the Japan Veterinary Medical Association [27]. The sex of the captured raccoons was determined prior to dissection based on their genitalia. During the study period, 252 raccoons were captured, of which 155 were male, and 97 were female. Age categories were determined based on tooth eruption [28] and cranial suture closure [29] after the removal of the skin and tissues around the skull. Raccoons were classified into five age categories: under 4 months, 5–11 months, 12–17 months, 18–23 months, and older than 24 months.

### 2.2. DNA Extraction

The kidney and urine of individual racoons were collected, and the samples were frozen at −30 °C. The kidneys were thawed, cut into 5 mm squares in a safety cabinet, placed in tubes, and 100 µL of sterile saline was added to the tubes. After shredding the sample in the tubes, additional 900 µL of sterile saline was added and mixed by vortexing. The sample was centrifuged at 800× *g* for 5 min to precipitate large organ fragments. The supernatant was transferred to a new tube and centrifuged at 15,000× *g* for 5 min. The resulting supernatant was used for DNA extraction. The urine samples were usually collected from the bladder; if the sample could not be collected in this way, 5 mL sterile saline was injected into the bladder, and 2 mL of the sample was then collected. Urine samples were centrifuged at 15,000× *g* for 5 min. The supernatant from the kidney and urine samples was removed and 200 µL of Insta Gene TM Matrix (DNA Purification Kit, Tokyo, Japan) was added. DNA was extracted using the Insta Gene TM Matrix protocol. The DNA was purified using the phenol–chloroform method. An equal amount of phenol/chloroform/isoamyl alcohol was added to the DNA samples and vortexed. The sample was centrifuged at 14,000× *g* for 5 min and the supernatant was transferred to a new tube. Equal amount of 2-propanol and 1/10 volume of 3M sodium acetate was added, and the mixture was vortexed. The sample was centrifuged at 20,800× *g* for 5 min and the supernatant was removed. The sample was washed with 500 µL of 70% Ethanol. After the supernatant was removed, it was dissolved in 100 µL 1× TE buffer.

### 2.3. Polymerase Chain Reaction (PCR) for Leptospira Detection

PCR was performed to confirm the presence of pathogenic *Leptospira*. Following a previous study [30,31] with modification, a 242 bp fragment of LipL32 present in the outer membrane lipoprotein of pathogenic *Leptospira* was targeted using the primers LipL32-45F (5′-AAG CAT TAC CGC TTG TGG TG-3′) and LipL32-286R (5′-GAA CTC CCA TTT CAG CGA TT-3′). The PCR mixture consisted of 5.0 µL of the extracted DNA sample, 10× PCR buffer, 200 µM dNTP mixture, 500 nmol/L of each primer, 0.5 U EX Taq (Takara, Shiga, Japan) in 20 µL. The reaction conditions were as follows: PCR initial heat denaturation at 95 °C for 2 min, 45 cycles of heat denaturation at 94 °C for 1 min, annealing at 50 °C for 1 min and, elongation reaction at 72 °C for 1.5 min, and final elongation reaction at 72 °C for 10 min. The PCR products were confirmed using 2.0% agarose gel electrophoresis.

### 2.4. Enzyme-linked Immunosorbent Assay (ELISA) for Anti-leptospiral Antibody Detection

Serum samples were collected from captured raccoons and used for ELISA. The Novivac Lepto vaccine (inactivated canine leptospirosis vaccine, Tokyo, Japan) was used as the ELISA antigen. Antigen was diluted 1:800 with 0.05 M carbonate buffer (pH 9.6), and 50 µL of antigen was coated onto a microplate (ELISA plate, Osaka, Japan) overnight at 4 °C. After washing with washing buffer (phosphate buffered saline with tween 20 [PBS-T]), 100 µL of blocking buffer (3% skim milk [As One, Osaka, Japan]) was injected into each well and allowed to react for two h at 37 °C. After washing with washing buffer, 50 µL of serum diluted 1:100 in 3% skim milk was injected into each well and allowed to react for one h at 37 °C. PBS-T was added to certain wells as a blank. After washing with washing buffer, 50 µL of anti-raccoon IgG serum (Alpha Diagnostic International, San Antonio, TX, USA) diluted 1:5000 with 3% skim milk was injected into each well and allowed to react for one h at 37 °C. After washing with washing buffer, 100 µL of 1:1 mixture of ABTS (2, 2´-azino-bis (3-Ethyl-benzothiazoline-6-Sulfonic Acid)) substrates A and B (SeraCare Life Sciences, Inc., Milford, CT, USA) was added to each well. After shaking the microplate in the dark for 30 min, 100 µL chromogenic stopper solution (1% sodium dodecyl sulfate) was added to each well. The absorbance was measured at 405 nm using a microplate reader. The difference between the absorbance (optical density [OD]) of the samples and the blank was determined. We used the average OD value obtained from three measurements to account for experimental error. As a positive control, one raccoon kept in zoos was vaccinated twice with Novivac Lepto at 4 and 2 week intervals. A positive result was assumed when the average OD value of each well was more significant than or equal to the average OD value of the positive control. 

### 2.5. Environmental Factors

The results of PCR and ELISA in raccoons were examined for their association with several environmental factors, including season, average monthly temperature, average rainfall, raccoon population density, urban area, and total water resource extension. 

Season: seasonal divisions were defined as spring, from March to May; summer, from June to August; fall, from September to November; and winter, from December to February.

Temperature and Rainfall: average monthly temperature and rainfall was determined based on Japan Meteorological Agency.

Population density: The raccoon population density was determined using capture per unit effort (CPUE), a measure of wildlife population density. CPUE is the number of raccoons captured per 100 capture effort based on the total capture effort (trap duration × number of trap units installed).

Urban area and Water resource: a circular buffer with a 400 m radius centered on the capture location was created, and overlapping areas were fused. The buffer was used for the capture area to estimate environmental factors. The radius of the circular buffer was the same as that used in a previous study [32] that estimated the association between raccoon infection and their habitat environment. The urban area (urban area in buffer/ buffer area (km^2^)) was determined based on high-resolution land cover maps provided by the Japan Aerospace Exploration Agency (JAXA). We followed a previously published method [33] for total water resource extension in this study. Based on the basic map information provided by the Geospatial Information Authority of Japan (GSI) of the Ministry of Land, Infrastructure, Transport, and Tourism, this study used the water horizon as the water resource. It calculated the total water resource length (km) within the circular buffer. QGIS (version 3.24.0.2, https://www.npackd.org/p/qgis64/3.24.0.2, accessed date 19 December 2022) was used for spatial analysis to obtain the data for the above-mentioned analysis.

### 2.6. Statistical Analysis

To compare leptospiral DNA and antibody detection status based on sex and age, we compared the results of PCR and ELISA. Univariable logistic regression was used to investigate the environmental factors associated with pathogenic *Leptospira* and anti-leptospiral antibodies in raccoons. We used the results obtained from the PCR and ELISA results as response variables. The seasons in which raccoons were captured, average monthly temperature, average monthly rainfall, the raccoon population density of the capture site, the urban area ratio of the capture site, and the water resource length ratio of the capture site were used as the predictor variables. Descriptive and inferential statistics were computed using R (version 4.2.2) (R core team, Vienna, Austria). Statistical significance was set at *p* < 0.05.

## 3. Results

**Figure 2:**
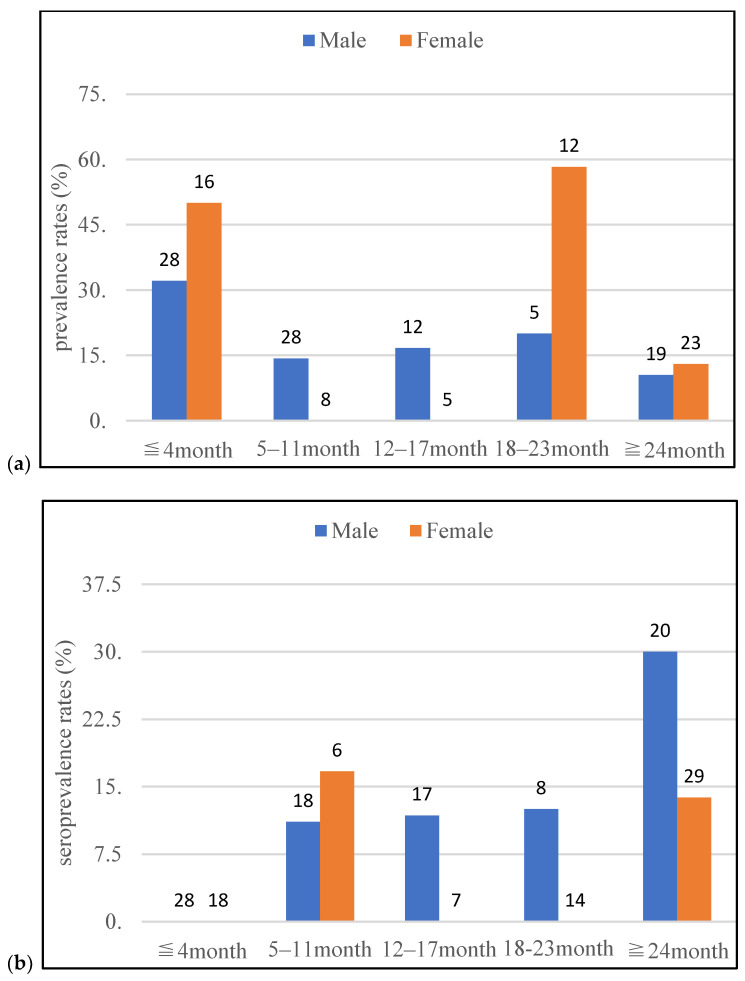
The leptospiral prevalence (**a**) and seroprevalence (**b**) rates, classified based on sex and age, in raccoons investigated in this study from June 2020 to April 2022 in Tama area, Tokyo, Japan (Number on the graph indicates the number of tested raccoons).

During the study period, 208 raccoons were used for either PCR or ELISA, of which 156 raccoons were used for PCR, and 165 raccoons were subjected to antibody detection. Through PCR, 36 raccoons (23.1%) were positive for pathogenic *Leptospira* in either the kidney or urine. Leptospiral DNA was detected in 31 kidney (19.9%) and 7 urine (4.5%) samples. Through ELISA, 16 raccoons (9.7%) tested positive for anti-leptospiral antibodies. The results of PCR and ELISA, classified according to sex, age, season, and capture area, are shown in Table 1. The prevalence and seroprevalence rates classified by sex and age are shown in Figure 2. Male raccoons between the ages of 5–11 and 12–17 months tended to have higher prevalence rates than females, and females between the ages of 18–3 months tended to have higher prevalence rates than males. By contrast, male raccoons between the ages of 12–17 and 18–23 months tended to have a higher seroprevalence rates than females. Male raccoons above the age of 24 months also tended to have a higher seroprevalence rates than females. However, female raccoons above the age of 24 months tended to have higher seroprevalence rates than females between the ages of 12–17 and 18–23 months. And male raccoons tended to be similar to the female raccoons.

The results of univariate logistic regression analysis are shown in Table 2. Significant differences were observed in PCR based on seasons (spring-summer) (95% CI 2.42–152.33, *p* = 0.01), average monthly temperature (95% CI 1.15–1.41, *p* < 0.01), and average monthly rainfall (95% CI 1.01–1.02, *p* < 0.01). There was no significant difference in the ELISA results, but raccoons living in larger urban areas tended to have higher seroprevalence rates (95% CI 0.89–819.7, *p* = 0.06). No significant differences in population density or water resources were observed between PCR and ELISA.

## 4. Discussion

Based on our observations from this study, pathogenic leptospiral prevalence and seroprevalence rates in raccoons differed according to sex and age. The late juvenile dispersal period of raccoons varied according to their sex. Migration during the late juvenile dispersal period provides an opportunity to be in contact with the environment apart from the natal area. It increases the possibility of exposure to environments contaminated with *Leptospira*. Male raccoons were reported to disperse at around five months, which is earlier than that observed for females [22,23]. Therefore, male raccoons end their dispersal period earlier than females. The higher prevalence rates in males aged 5–11 and 12–17 months than that observed in females could be owing to an earlier dispersal period in males than in females. As the seroprevalence rates in this study indicates a history of infection, the differences in the prevalence rates based on the dispersal period may be reflected later in the seroprevalence rates. As a result, male raccoons between the ages of 12–17 and 18–23 months may have a higher seroprevalence rates than females.

By contrast, female raccoons are expected to remain in their natal area after the males disperse, and their dispersal period is known to be later than that of males [23]. Therefore, female raccoons end their dispersal period later than male raccoons. The prevalence rates may be reversed according to the sex at 18–23 months, which is later than the age of 5–11 and 12–17 months at which males have higher prevalence rates than females. Although there was no reversal, according to the sex observed in the seroprevalence rates, above the age of 24 months in the seroprevalence rates, and males and females above the age of 24 months may have higher seroprevalence rates than males and females between the ages of 12–17 and 18–23 months. Although no study has conducted an age assessment as detailed in this study, the results of this study may be similar to those of a study that showed that seroprevalence rates tends to be higher in adult raccoons when divided into juvenile and adult animals.

Further, male raccoons have a longer dispersal distance and a wider home-range than females [34,35,36]. Based on these facts, males are expected to have greater exposure to contaminated environments. Therefore, male raccoons above the age of 24 months showed a higher seroprevalence rates than females. Male behavioral characteristics may explain why reversal according to the sex in the seroprevalence rates was not observed above the age of 24 months. Additionally, young males may spread *Leptospira* via male dispersal. The differences in leptospiral prevalence and seroprevalence rates based on the raccoon dispersal period may indicate the pattern of pathogen spread by raccoons and may be an important finding linking the behavioral characteristics of wild animals to zoonotic infections. 

An accurate age assessment is required to investigate the route of infection through raccoon dispersal; however, this takes a considerable amount of time. Therefore, it should be noted that this study may not be generalizable to some age groups because of the small sample size or the number of positives animals. A further increase in the sample size is desirable for future studies. ELISA in this study is a simple measurement and may have underestimated the results in this study compared to the PCR results, since a limited number of serovars of vaccine antigens were used as ELISA antigens and vaccine-immunized raccoon were used as positive controls. Further improvement of measurement is needed, and the ELISA results were used to corroborate the PCR results.

Based on the PCR results, the prevalence rates in raccoons were high during seasons with high average monthly temperatures and rainfall. Several studies have shown that leptospirosis is associated with higher temperature in other animals apart from raccoons [37,38,39,40,41]. A correlation was observed between the occurrence of leptospirosis in dogs and temperature, indicating that climatic conditions contribute to the survival of *Leptospira* and wild rats and that climate changes brought about by global warming and other factors could potentially contribute to the increased occurrence of leptospirosis [39]. As summer and autumn in Japan are seasons with high average rainfall, *Leptospira* are most likely to spread during these seasons [42,43]. This study observed an association between leptospiral prevalence rates in raccoons and temperature and rainfall, suggesting that leptospiral prevalence rates in raccoons are associated with increased temperature and rainfall.

In the study of *Leptospira* in raccoons in California, USA, the study period was divided into a dry season, in which the temperature was high and rainfall was low, and a wet season, in which the temperature was low and rainfall was high. As a result, the prevalence rates in raccoons were found to be high in the wet season, suggesting that the wet season may lead to a highly moist environment suitable for the survival of *Leptospira* [44]. It is possible that rainfall may have a stronger effect on transmission than temperature; however, this is difficult to clearly assess in the Japanese climate, which is not divided into dry and wet seasons, and temperature and rainfall tend to be linked. To determine whether temperature or rainfall has a stronger influence on prevalence rates, a multi-year study, including periods when temperatures and rainfall are not linked, is needed.

In this study, the prevalence rates of raccoons living in the suburbs of Tokyo were equal to or greater than those of raccoons from other areas of Japan [18,20,21]. Our results suggest raccoons in urban areas with high population densities may also carry *Leptospira*. However, the primers used in the surveys in other region differed, and the same was true in this study. Since differences in sensitivity due to primers have been noted [18,31], caution should be exercised when interpreting prevalence rates. Although there were no significant differences, our study is the first to quantify and estimate the environmental factors that affect leptospiral prevalence and seroprevalence rates in raccoons. There are urban species of wild rodents, with Norvegicus rats (*Rattus norvegicus* [Berkenhout 1769]) and black rats (*Rattus rattus* [Linnaeus 1758]) being the most common. Norvegicus and black rats are the prime sources of *Leptospira* in comparison with other rat species [45] and *Leptospira* have been isolated from Norvegicus rats found in Tokyo [46]. The results of this study suggest that raccoons living in larger urban areas may be in contact with Norvegicus and black rats and share the same habitat. However, analysis of species relevance except for raccoons, is needed because our study did not investigate wild rodents.

In urban areas, raccoons have been recognized for their role as sentinels for several pathogenic agents. *Leptospira* are no exception, and it has been suggested that raccoons may introduce serovars that are not confirmed in the area and function as a part of the infection cycle for such serovars [16,47]. Therefore, studies pertaining to *Leptospira* serovars can reveal the infection cycle in urban wildlife, including raccoons and wild rodents. Tokyo is the most populous city in Japan; however, its suburbs are adjacent to green and residential areas. It has been suggested that medium-sized wild animals, including raccoons, come to resident areas from green areas, and wildlife habitats are close to human living areas [48]. As a result, raccoons adaptable to urban areas are more likely to make urban areas a part of their home-range and may spread *Leptospira*.

Although no significant differences were observed in terms of population density and water resources in PCR and ELISA, these may be important environmental factors to consider in studies of infectious diseases and wildlife. As the sample size and study area required for estimating environmental factors were not sufficient in this study, it is possible that there were no significant differences in terms of population density and water resources. The importance of these environmental factors needs to be assessed in future studies.

## 5. Conclusions

Leptospirosis is a zoonotic infection prevalent globally in humans and animals. Several outbreaks have been reported in Japan, making this a significant public health concern. It is also widespread among wild animals, as it is found in almost all mammals. Wild animals that pose an infection risk to humans are those that make human living areas part of their home-range. The raccoon, an invasive species whose population and distribution are expanding in Japan, is one such animal. This study showed that raccoon behavior pattern (dispersal period) that differ by sex and age affect the prevalence and seroprevalence rates, revealing the pattern of leptospiral spread in raccoons. The same may be true for other infections and wildlife with different behavioral characteristics based on sex and age. Further studies are needed to clarify the patterns of pathogen spread based on sex and age.

No significant difference was observed in ELISA results; however raccoons living in larger urban areas tended to have higher seroprevalence rates. Therefore, we suggest that raccoons may be associated with rats, that inhabit urban areas and are an important source of *Leptospira*. This study is the first to quantify and estimate environmental factors related to leptospiral prevalence and seroprevalence rates in raccoons.

## Figures and Tables

**Table 1:** Positive cases were observed during PCR and ELISA, classified in various categories, in raccoons investigated in this study from June 2020 to April 2022 in the Tama area, Tokyo, Japan.

		PCR			ELISA		
		Test	Positive	%	Test	Positive	%
Sex	Male	92	18	19.6	91	11	12.1
	Female	64	18	28.1	74	5	6.8
Age (months)	≤4	44	17	38.6	46	0	0
	5–11	36	4	11.1	24	3	12.5
	12–17	17	2	11.8	24	2	8.3
	18–23	17	8	47.1	22	1	4.5
	≥24	42	5	11.9	49	10	20.4
Seasons	Spring	25	1	4.0	41	6	14.6
	Summer	54	24	44.4	53	7	13.2
	Autumn	61	11	18	53	3	5.7
	Winter	16	0	0	18	0	0
Capture Area	A	36	8	22.2	41	1	2.4
	B	32	8	25	-	-	-
	C	19	5	26.3	22	2	9.1
	D	9	0	0	5	1	20
	E	15	4	26.7	15	4	26.7
	F	9	1	11.1	21	3	14.3
	G	8	0	0	12	1	8.3
	H	28	10	35.7	41	4	9.8
	I	-	-	-	6	0	0
	J	-	-	-	2	0	0
	Total	156	36	23.1	165	16	9.7

**Table 2:** Univariate logistic regression analysis estimates the factors responsible for leptospiral prevalence and seroprevalence rates in raccoons investigated in this study from June 2020 to April 2022 in Tama area, Tokyo, Japan.

Response Variable	Predictor Variable		OR	95% Cl	*p*
PCR (n = 156)	Seasons	Spring (n = 25)	-		
		Summer (n = 54)	19.2	2.42–152.33	0.01
		Autumn (n = 61)	5.28	0.64–43.3	0.12
		Winter (n = 16)	NA	NA	NA
	Temperature		1.27	1.15–1.41	<0.001
	Rainfall		1.01	1.01–1.02	<0.001
	Population density		1.08	0.97–1.21	0.18
	Urban area		1.30	0.40–4.25	0.66
	Water resource		1.16	0.83–1.63	0.39
ELISA (n = 165)	Seasons	Spring (n = 41)	-		
		Summer (n = 53)	0.89	0.27–2.88	0.84
		Autumn (n = 53)	0.35	0.08–1.49	0.16
		Winter (n = 18)	NA	NA	NA
	Temperature		1.03	0.95–1.12	0.45
	Rainfall		1.0	0.99–1.006	0.92
	Population density		0.91	0.76–1.10	0.34
	Urban area		27.0	0.89–819.7	0.06
	Water resource		0.73	0.49–1.10	0.13

OR: odds ratio, 95% CI: 95% confidence interval, NA: not analyzed.

## Data Availability

The data used in the analysis are available from the authors on request.
